# Phytochemical Characterization and Bioactivity of *Asparagus acutifolius*: A Focus on Antioxidant, Cytotoxic, Lipase Inhibitory and Antimicrobial Activities

**DOI:** 10.3390/molecules26113328

**Published:** 2021-06-01

**Authors:** Amel Hamdi, Sara Jaramillo-Carmona, Rocío Rodríguez-Arcos, Ana Jiménez-Araujo, Mokhtar Lachaal, Najoua Karray-Bouraoui, Rafael Guillén-Bejarano

**Affiliations:** 1 Phytochemicals and Food Quality Group, Instituto de la Grasa (CSIC), 41013 Seville, Spain; amelhamdi1988@yahoo.fr (A.H.); smjaramillo@ig.csic.es (S.J.-C.); rrodri@ig.csic.es (R.R.-A.); araujo@ig.csic.es (A.J.-A.); 2 Unité de Physiologie et de Biochimie de la Réponse des Plantes aux Contraintes Abiotiques, FST Campus, Université Tunis El Manar, 1068 Tunis, Tunisia; mokhtar.lachaal@fst.rnu.tn (M.L.); najouakarraybouraoui@yahoo.fr (N.K.-B.)

**Keywords:** *Asparagus acutifolius*, phytochemicals, antioxidant activity, lipase inhibitory activity, cytotoxicity, antimicrobial activity, LC-MS, HPLC-DAD, HPLC/Q-TOF-MS

## Abstract

The phytochemical composition of leaves, stems, pericarps and rhizomes ethanolic extracts of *Asparagus acutifolius* were characterized by HPLC-DAD-MS. *A. acutifolius* samples contain at least eleven simple phenolics, one flavonon, two flavonols and six steroidal saponins. The stem extracts showed the highest total phenolic acid and flavonoid contents, where cafeic acid and rutin were the main compounds. No flavonoids were detected in the leaf, pericarp or rhizome while caffeic acid and ferulic acid were the predominant. Steroidal saponins were detected in the different plant parts of *A. acutifolius*, and the highest contents were found in the rhizome extracts. The stem extracts exhibited the highest antioxidant activity against 2,2-diphenyl-1-picrylhydrazyl (DPPH) and ferric reducing antioxidant power (FRAP) and the highest 2,2-azino-bis (3 ethylbenzothiazoline-6-sulphonic acid) (ABTS) scavenging activity was found in the pericarp extracts. The rhizome and leaf extracts showed a potent cytotoxic activity against HCT-116 and HepG2 cell lines. Moreover, the pericarp and rhizome extracts revealed a moderate lipase inhibitory activity. The leaf and rhizome extracts were screened for their antimicrobial activity against human pathogenic isolates. The leaf extract exhibited a powerful inhibitory activity against all the bacteria and fungi tested.

## 1. Introduction

In recent decades, an increasing interest has been focused on wild edible plants to broaden the diversity of the human diet because of their nutritional and medicinal values [1,2]. In the Mediterranean basin, wild plants rich in antioxidants have been harvested and eaten seasonally for many generations. They have been used as food, medicine, dye and ornaments and are also an important source of income for local people in different regions. Among edible wild plants, the *Asparagus* genus has a relevant position, including over 250 species of both food and medicinal interest [3]. Among these, *Asparagus acutifolius* L., is an herbaceous, perennial species widely distributed in the Mediterranean area, and mainly known for the fine flavor of its spears. It is generally sold in local markets at relatively high prices and used in typical restaurant dishes. From the nutritional point of view, *A. acutifolius* spear, which is the edible part of the plant, is rich in antioxidant phenolics, Vitamin C, folates, ascorbic acid and dietary fiber [4]. Among phenolics, the major compounds found are Quercitin-3-*O*-rutinoside and Isorhamnetin-3-*O*-rutinoside and to a lesser extend Kaempherol-3-*O*-rutinoside [5]. Aqueous extracts of *A. acutifolius*, rich in these compounds, have been shown to possess significant antiproliferative and pro-apoptotic activity against bladder and lung cancer lines [6]. Di maro et al. [7] have found rutin (3-*O*-Quercitin-rutinoside) as the main flavonoid along with high levels of isoquercitrin (Quercitin-glucoside). They have also found important antiproliferative activities against neuroblastoma and liver, cervical and lung cancer cells. This plant is also used for its medicinal properties which are related to the presence of different bioactives in various plant parts including phenolics, saponin and polysaccharides [8]. For instance, a decoction of the root has been used traditionally by the ethnomedicine as a diuretic [9].

However, in recent years, wild edible food sources, particularly wild *A. acutifolius*, have fallen into neglect since their traditional uses are being lost. The revalorization of these wild vegetables as potential sources of functional food ingredients and/or including their consumption in modern diets could contribute to the preservation of their traditional knowledge and culinary uses, as well as to improve the biodiversity and rural sustainability of the collection areas.

Among plant secondary metabolites, phenolic compounds have been extensively studied and are commonly used as antioxidants because they might retard the oxidative degradation of lipids and thereby improve the quality and nutritional value of food [10]. Asparagus contains flavonoids (mainly rutin) and other phenolic compounds which possess strong antioxidant properties [11,12,13,14]. The potential health benefits of the antioxidants in asparagus include reducing the risk of cancer, cardiovascular and cerebrovascular diseases [15,16,17].

As far as we know there is no report on either the biological activity or the phytochemical composition of the leaf, stem, pericarp or rhizome of *A. acutifolius*. Except for a report on the characterization of the root’s saponin [8], the available studies in the literature describe the nutritional assessment, phenolic composition and antioxidant properties of only the edible portion of this plant [18,19,20]. However, considering the presence of different compounds in different portions of the plant, it seems interesting to study the different organs as possible sources of bioactives with different types of applications in sectors such as food, cosmetics or pharmaceuticals. Therefore, the aim of this study was to characterize the bioactive compounds of the *A. acutifolius* leaf, stem, pericarp and rhizome, paying special attention to the functional properties (antioxidant, cytotoxic, lipase inhibitory and antimicrobial activities) of their ethanolic extracts.

## 2. Results and Discussion

### 2.1. Analysis of Phenolic Compounds

A total of 16 different phenolic compounds were identified and quantified in the ethanolic extracts of different plant parts of *A. acutifolius* (Table 1).

In particular, seven components were recognized as p-hydroxybenzoic acid derivatives (vanillic acid, *p*-hydroxybenzaldehyde, *p*-hydroxybenzoic acid, vanillin, syringic acid, protocatechuic acid, and gallic acid), four as hydroxycinnamic acids (ferulic acid, caffeic acid, *p*-coumaric, and sinapic acids) and five as flavonoids (naringenin, quercetin, kaempferol, rutin and narcissin). The profiles of phenolic compounds varied markedly among the samples. The results showed that the total content of simple phenolics (*p*-hydroxybenzoic acid derivatives and hydroxycinnamic acids) was 1.3, 1.9 and 2 times higher in the stem than in the rhizome, pericarp and leaf, respectively (Table 1). In the different plant parts, caffeic acid was the predominant phenolic acid, and its value was 72% of the total simple phenolics quantified in the stem, 31% in the pericarp, 19% in the leaf and 16% in the rhizome extracts (Table 1). The results of our analysis are in accordance with those of Salvatore et al. [18], who found that caffeic acid was the main phenolic acid in *A. acutifolius* spear and A. officinalis root. In addition to caffeic acid, significantly higher amounts of other simple phenolics (*p*-hydroxybenzaldehyde, *p*-hydroxybenzoic, vanillic, syringic, coumaric, ferulic and sinapic acids) were found in the rhizome extract compared to the other organs (Table 1). Different results were found in the work of Lake et al. [21] and Symes et al. [22], which showed that vanillic and ferulic acids were not identified in the root of A. officinalis. However, Billau et al. [23] observed only low levels of vanillic acid and did not detect ferulic acid in storage roots of white asparagus. Concerning flavonol glycoside, significant quantities of rutin and narcissin were quantified from the stem, but they were not detected in the leaf, pericarp or rhizome (Table 1). Barros et al. [19] also reported that the flavonoid profile of the young stems of *A. acutifolius* from Portugal was comprised of rutin, narcissin and nicotiflorin. In addition, the rhizome was richer in flavonols (quercetin and kaempferol) than the other extracts. Naringenin was the only flavanone quantified, being present in the highest amount in the stem. This flavanone has also been detected and isolated from Asparagus sprengeri [24].

The results of this study showed statistically significant differences in the content of total phenolics among wild *A. acutifolius* organs. More than 80% of the total amount quantified is simple phenolics in the leaf, rhizome and pericarp, but only 44% in the stem, where flavonoids were the most abundant.

### 2.2. Saponins in the Different Plant Parts of A. acutifolius

Six saponins were identified in the sample of *A. acutifolius*, three of them (HTSAP-1, HTSAP-3 and HTSAP-6) were previously identified in triguero HT [25,26,27] and other three saponins were identified as new structures (from ACSAP-1 to ACSAP-3). Those saponins were identified by their retention time, molecular ion (*m*/*z*), and fragmentation pathway. The fragmentation pathway has been studied through the mass spectrum obtained in negative (100 V−) and positive (50 V+) modes (Table 2).

ACSAP-1 showed the same molecular ion and pathway fragmentation model as that reported for HTSAP-1 [25] but presented a different retention time (Table 2). This saponin was detected in the different plant parts of *A. acutifolius* except in the rhizome. ACSAP-1 exhibited a quasi-molecular ion peak in negative ion mode [M-H]- at *m*/*z* 1051 and molecular formula C_50_H_83_O_23_. Other fragment ion peaks can be seen at *m*/*z* 919, 757, 595 and 433, resulting from consecutive losses of one pentose and three hexoses. In the case of the positive spectrum mode, it showed the ions at *m*/*z* 1075 (sodium adduct), *m*/*z* 1035 (loss of one H_2_O molecule), and *m*/*z* 873, 711, 579 and 417 corresponding to the loss of a hexose, a hexose, a pentose and a hexose, respectively.

In addition, two new saponins (ACSAP-2 and ACSAP-3) were identified only in the rhizome extract of A. *acutifolius* in very small amounts, being 3% and 5% of the total saponin contents, respectively. According to the molecular weight and fragmentation pathway of ACSAP-2 and ACSAP-3 summarized in Table 2, those saponins could be similar to those described in the root of A. *acutifolius* by Sautour et al. [7], where both of them have a hydroxysarsapogenin as aglycon. In our study, the negative mode of ACSAP-2 showed an ion fragment at *m*/*z* 887 [M-H]- and the molecular formula C_44_H_71_O_18_. Ion peaks at *m*/*z* 755, 593 and 431 were also observed, which correspond to the loss of two hexoses and one pentose. The positive mode spectrum showed the ions at *m*/*z* 911 (adduct where a molecule forms with sodium), *m*/*z* 871 (loss of one H_2_O molecule), and *m*/*z* 709, 577, and 415, which correspond to the loss of a hexose, a pentose and a hexose, respectively.

ACSAP-3 in positive and negative mode was compatible with a saponin containing two pentoses and one hexose. In negative ion mode, an ion peak at *m*/*z* 857. [M-H]-, indicating a molecular formula of C_43_H_69_O_17_, was observed. Other ion peaks were also observed, such as *m*/*z* 725, 593 and 431, which correspond to the loss of a pentose, a pentose and a hexose respectively. In the case of the positive spectrum mode, it showed ions at *m*/*z* 881 (adduct where a molecule forms with sodium), *m*/*z* 841 (loss of one H_2_O molecule), and *m*/*z* 709, 577 and 415, which correspond to the loss of a pentose, a pentose and a hexose, respectively.

**Figure 1:**
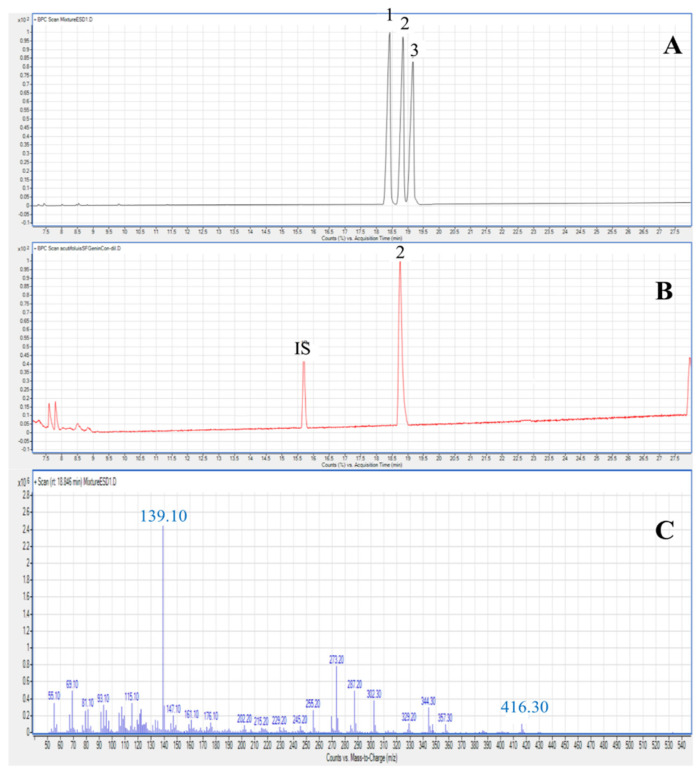
Chromatographic profile acquired by GC-MS of genin standards (**A**), hydrolyzed acid of saponin fraction of *A. acutifolius* organs (**B**) and mass spectra of sarsasapogenin (**C**). 1: Esmilagenin, 2: Sarsasapogenin, 3: Diosgenin, IS: Internal standard cholesterol.

In order to confirm the genin of the saponins previously described in our study, a GC/MS method was used. Steroidal sapogenins have been obtained by acid hydrolysis of the crude saponin fraction and the major saponins isolated from the different organs of A. *acutifolius*. The ESI-MS obtained during GC/MS were characterized by the appearance of the base peak at *m*/*z* 139 (Figure 1C). Several other diagnostic ions were also observed, which coincide with others previously described by Sauvaire et al. [28]. The identification of sarsasapogenin (peak 2, M^+^ 416) was also based on the comparison of retention times and mass spectra to those of authentic standards of these sapogenins (Figure 1A,B).

**Figure 2:**
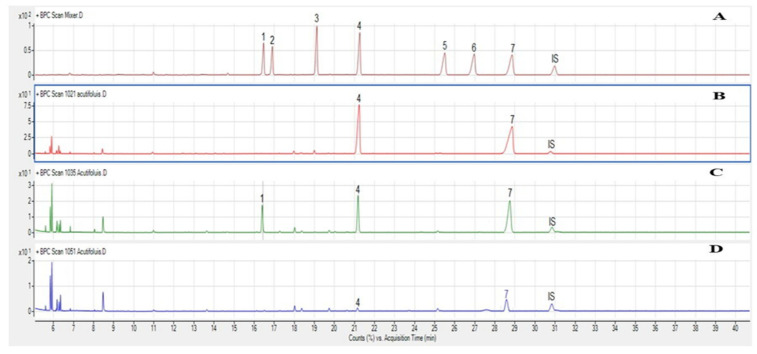
Chromatographic profile acquired by GC-MS of sugar standards (**A**), the major saponins detected in the hydrolyzed acid of *A. acutifoius* organs; (**B**), HTSAP-3, (**C**), HTSAP-6, (**D**), HTSAP-3 and ACSAP-1. 1: Rhamnose, 2: Fucose, 3: Arabinose, 4: Xylose, 5; Mannose, 6: Galactose, 7: Glucose, IS: Inter- standard; Inositol.

This genin was the major one found in our samples as it is commonly found in the *Asparagaceae* family [7,8,9,10,11,12,13,14,15,16,17,18,19,20,21,22,23,24,25,26,27,28,29]. The glycosidic composition was also analyzed in order to obtain more information about the structural characteristics of the identified saponins, especially the major ones such as HTSAP-1, ACSAP-1, HTSAP-3 and HTSAP-6. The monosaccharides obtained after the hydrolysis of the major saponins isolated from *A. acutifolius* were glucose and xylose in HTSAP-1, ACSAP-1 and HTSAP-3 (Figure 2B,D). However, glucose, xylose and rhamnose were identified in HTSAP-6 (Figure 2C).

The saponins have also been quantified in the ethanolic extracts from the different plant organs by the external standard method (Table 3). The results show that the total content of saponins in the rhizome were 3, 5 and 24 times higher than in the pericarp, leaf and stem, respectively. The leaf, pericarp and stem mainly contained HTSAP-6, which represent 75%, 63% and 59% of their total contents, respectively. A high amount of the new saponin (ACSAP-1), which represented 12%, 16% and 25% of the total saponin contents in the leaf, stem and pericarp extracts, respectively, was also detected. The ethanolic extract from the rhizome was mainly comprised of three saponins, as previously described in triguero HT asparagus, HTSAP-3 (47%), HTSAP-1 (24%) ad HTSAP-6 (21%) [25]. Two minor peaks were also identified in the rhizome extract, as previously described by Sautour et al. [7] (ACSAP-2 and ACSAP-3) or described in this work as tentative new saponins based on the mass spectra. ACSAP-2 and ACSAP-3 present only 3% and 5%, respectively, of the total saponin contents in the rhizome extracts of *A. acutifolius*.

Among the different parts of *A. acutifolius*, the content of each saponin was always the highest in the rhizome, followed by the pericarp, leaf and then stem. This observation was consistent with previous studies [30,31]. In accordance with our previous work [31], a high content of saponins was also detected in the rhizome of *A. albus* compared to the leaf and pericarp, probably due to the need for protection against pathogen attacks in the soil. Working with a saponin-deficient oat mutant, Papadopoulou et al. [32] concluded that saponins may have general significance as antimicrobial phytoprotectans.

In summary, both quantitative and qualitative studies suggested that the rhizome was significantly different from the other parts. It contained the highest contents of individual saponin and total saponins, clearly pointing to this part as a raw material for the manufacture of saponin-based products. The leaf and stem also showed clear differences and contained the lowest contents of saponins. Different results were reported for *Bacopa monnieri*, in which the highest content of saponins was found in the leaf and shoot compared to the root [33]. These differences between organs could also justify the differential functional properties which have been attributed to the different plant parts from ancient times.

### 2.3. Evaluation of Antioxidant Capacity

**Figure 3:**
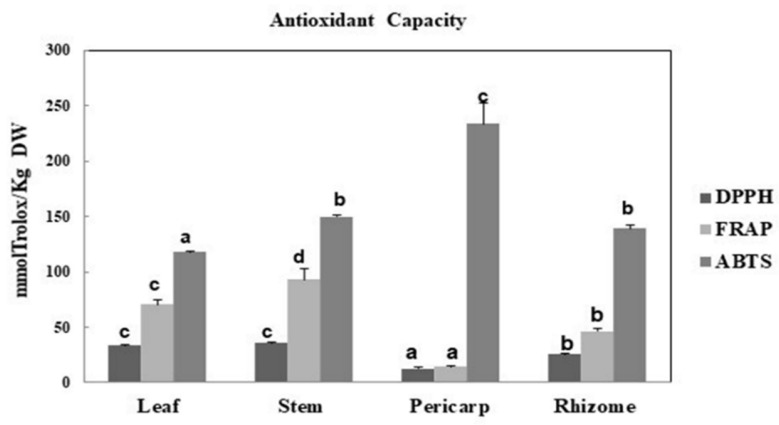
Antioxidant activities, expressed as mmol Trolox equivalent/Kg of dry weight in *A. acutifolius* leaf, stem, pericarp and rhizome ethanolic extracts, determined by DPPH, FRAP and ABTS assays. DPPH: DPPH radical scavenging activity; FRAP: Ferric reducing antioxidant power; ABTS: ABTS radical scavenging activity. Results correspond to the mean ± standard deviation of three replicates. Different letters (a–d) indicate that the values are significantly different (*p* < 0.05). The comparison is for different samples with the same method.

In the current study, for the determination of antioxidant capacity, ABTS, FRAP and DPPH assays were performed. The results obtained from the three methods are shown in Figure 3. For DPPH and FRAP assays, leaf and stem extracts exhibited the highest values and pericarp the lowest. Surprisingly, in the ABTS test, the pericarp was the most active extract. In the literature, most of the research on the antioxidant activity of *A. acutifolius* extracts was carried out on extracts obtained from the shoots, which are the edible parts [17,18,19,20]. However, its different organs (leaf, stem, rhizome and fruit) have no commercial value and very little research has been carried out on them. In a previous work, our research group characterized the same plant parts from *A. albus* [31]. In that case, similar results were found for the rhizome, but not for the pericarp or leaf, which also presented a very different composition of phytochemicals.

### 2.4. Cytotoxic Activity

**Figure 4:**
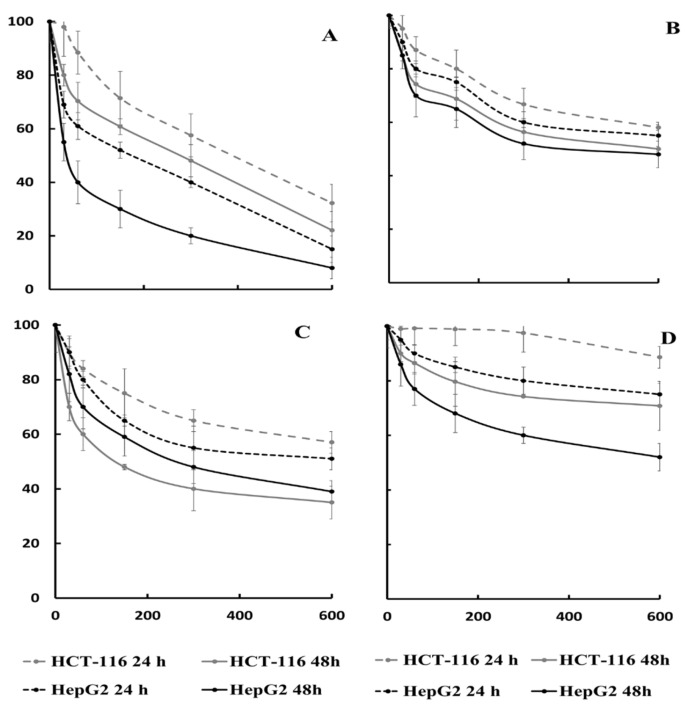
Cell viability assay on colon cancer (HCT-116) and human hepatocellular carcinoma (HepG2) cells after 24 and 48 h incubation with serial concentrations of the different organs’ ethanolic extracts from *A. acutifolius*. (**A**) Rhizome, (**B**) Stem, (**C**) Leaf and (**D**) Pericarp. The effect was measured by the MTT cell viability assay and the cytotoxic activities expressed as IC_50_ μg/mL of dry weight. Results correspond to the mean ± standard deviation of three replicates.

The cytotoxic activity of the extracts from different plant parts was tested in two distinct human cancer cell lines, colon cancer (HCT-116) and liver cancer (HepG2) lines, using the MTT assay, a method for screening anti-proliferative agents. Figure 4 shows cell viability dose-response curves for each extract after cells were incubated for 24 and 48 h.

The rhizome extract was cytotoxic in the two cell lines tested. The concentration producing 50% cell death (IC_50_) on HCT-116 cells was 388.5 µg/mL for 24 h and 245.14 µg/mL for 48 h (Figure 4). Surprisingly, the leaf extract was more cytotoxic than the rhizome for 48 h (IC_50_ 116.54 µg/mL) (Figure 4). The stem and pericarp extracts did not show cytotoxic activity in the tested conditions. For the HepG2 cell line, the rhizome had a very important cytotoxic effect at 48 h (IC_50_ 45.14 µg/mL), but the leaf also showed a moderate effect (IC_50_ 315.20 µg/mL) at 48 h (Figure 4).

Several studies have shown that saponins from asparagus are associated with a reduced risk of colorectal cancer by inducing cytotoxicity and apoptosis [34,35]. The IC_50_ value described for the rhizome extract was better than the saponins extracted from by-products of *A. officinalis* in the same cell line (902 µg/mL) [16], probably due to a different saponin composition or also to synergistic effects with other phytochemicals presents in the *A. acutifolius* extract. However, lower IC_50_ values (40 µg/mL) were found when working with the rhizome extract of *A. albus*, while the leaf extracts from these two asparagus species had similar IC_50_ values for HCT-116 cells [31]. Likewise, the rhizome from *A. acutifolius* showed an important cytotoxic activity against the HepG2 cell line and similar values were reported by other authors for A. *adscendent* [36] and A. *filicinius* [37], who related this activity to the presence of saponins and their genins.

The remarkable cytotoxicity observed in the current study against two carcinoma cell lines suggests that the rhizome and leaf from *A. acutifolius* could be exploited as a potential source of cytotoxic compounds with putative anticancer potential.

### 2.5. Lipase Inhibitory Activity

**Figure 5:**
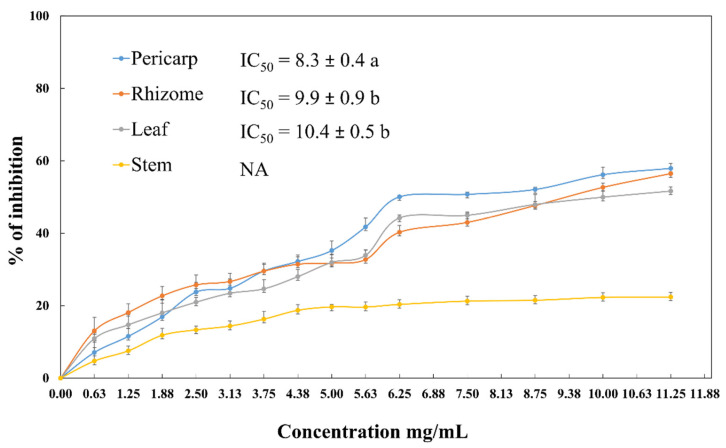
Pancreatic lipase inhibitory activity of leaf, stem, pericarp and rhizome ethanolic extracts of *A. acutifolius*. The pancreatic lipase inhibitory activity was expressed as IC_50_ μg/mL of dry weight. Results correspond to the mean ± standard deviation of three replicates IC_50_ values followed by different letters are statistically different. NA: no activity.

In the present study, the ethanolic extracts of *A. acutifolius* leaf, stem, pericarp and rhizome were evaluated for their inhibitory effect on porcine pancreatic lipase enzyme using an in vitro assay. As shown in Figure 5, *A. acutifolius* pericarp, rhizome and leaf extracts exhibited concentration dependent activity. The concentration which inhibited 50% of the lipase activity (IC_50_) was 8.3 mg/mL in the pericarp extracts, 9.9 mg/mL in the rhizome extracts and 10.4 mg/mL in the leaf extracts. However, no inhibition effect on the lipase activity was found in the stem extract.

Very little research has been carried out on in vitro lipase inhibitory activity in the different *Asparagus* species. However, previous studies have demonstrated the hypolipidaemic action of asparagus in animal models [38,39].

In previous studies, the administration of *triguero* HT spears to hypercholesterolemic rats improved the plasma lipid profile and prevented hepatic oxidative damage [15]. In addition, Visavadiya and Narasimhacharya [38], working with rats fed with a high-cholesterol diet, reported that the steroid-type saponins of *Asparagus racemosus* root seemed to be mainly responsible for the plasma cholesterol-lowering effect due to reduction in the absorption of cholesterol. The findings of Zhu et al. [39] also showed that the ethanolic and aqueous extracts from asparagus by-products had a strong hypolipidaemic effect and that they could be used as a supplement in healthcare foods and drugs or in combination with other hypolipidaemic drugs.

These results show that some *A. acutifolius* organs exhibited a moderate lipase inhibitory activity and they could be considered to use to improve the plasma lipid profile and to reduce the level of cholesterol.

### 2.6. Antimicrobial Activity

A preliminary essay showed that the leaf and rhizome ethanolic extracts of *A. acutifolius* exhibited a promising antimicrobial activity. For this reason, we screened those two organs for their inhibitory activities against 15 bacteria and 5 fungi isolated from various patients and the results are illustrated in Table 4.

The leaf ethanolic extracts showed a potent antimicrobial activity against all the Gram-positive and Gram-negative bacteria and the fungi tested with a zone of inhibition ranging from 14 to 22 mm for the bacteria and 13 to 27 mm for the fungi. The leaf extract showed the highest antibacterial activity against *Salmonella typhimurium* with an inhibition zone (22 mm) and against *Staphylococcus aureus* (MR) (18 mm), *Klebsiella pneumonia* (MR) (18 mm), *Escherichia cloacae* (18 mm), *Esclerichia faecalis* (17 mm), *Esclerichia faecium* (17 mm), *Listeria monocytogenes* (17 mm) and *Esclerichia coli* (17 mm).

Moreover, compared to the two antibiotics, gentamicin and streptomycin, leaf extracts showed better activity against *Listeria monocytogenes*, *Esclerichia faecalis*, and *Esclerichia coli* (ESBL). Compared to the leaf extracts, *Salmonella aureus*, *Listeria monocytogenes*, *Esclerichia coli* (BLSE) and *Proteus mirabilis* were not susceptible to the rhizome ethanol extracts; however, the later organ exhibited a higher activity only against *Esclerichia faecium* compared to the leaf extracts. The two organ extracts showed a higher activity against all the fungi than Amphotericin B. *Geotricum capitatum* was more susceptible to the leaf extracts than the rhizome extracts.

Nevertheless, the rhizome showed better activity against *Candida galabrata* than the leaf extracts. In accordance with our previous work [31], the leaf ethanolic extracts of *Asparagus albus* also exhibited a potent antimicrobial activity compared to the pericarp and rhizome extracts. In addition, the leaf ethanolic extracts of *A. acutifolius* showed a high antibacterial activity than the leaf ethanolic extracts of *A. albus*. However, the leaf and rhizome extracts of *A. albus* have higher antifugal activity against *Candida albicans* than those found in *A. acutifolius* [31]. The minimal inhibition concentrations (MICs) and the minimal bactericidal concentration (MBCs) values of *A. acutifolius* leaf and rhizome extracts tested against all strains are summarized in Table 5. The results indicate that the leaf was the most effective organ. In the two organs, the values of MICs and MBCs ranged from 0.195 to 3.12 mg/mL and from 3.125 to 50 mg/mL, respectively, against all strains. The ethanolic extracts of the leaf and rhizome were the most active against *Salmonella typhimurium* (for leaf extracts) and *Esclerichia faecium* (for rhizome extracts) with MIC of 0.195 mg/mL and MBC of 3.125 mg/mL.

Concerning yeasts, the results showed a concentration range of 0.096 to 0.390 mg/mL for MIC and 0.195 to 0.390 mg/mL for MFC in the two organ extracts and leaf ethanolic extracts showed greater antifungal activity against *Geotricum capitum* and *Candida parapsilosis* (MIC = 0.096 mg/mL and MFC = 0.195 mg/mL). However, the rhizome extract was most active against *Candida parapsilosis* (MIC = 0.096 mg/mL and MFC = 0.195 mg/mL).

## 3. Materials and Methods

### 3.1. Chemicals and Reagents

Authentic standards of quercetin, kaempferol and rutin (quercetin-3-*O*-rutinoside), naringenin, gallic acid, *p*-hydroxybenzoic acid, *p*-hydroxybenzaldehyde, vanillin, caffeic acid, *p*-hydroxyphenylacetic acid, ferulic acid, sinapic acid and 2,2-diphenyl-1-picrylhydrazyl (DPPH free radical), ferric chloride, 2,2′-dipyridyl (99% minimum purity), Trolox (97% purity), porcine pancreatic lipase, p-nitrophenyl butyrate (*p*-NPB), methylthiazolyldiphenyl-tetrazolium bromide (MTT), ethanol, formic acid (96%) and acetonitrile (HPLC grade), vanillic acid, *p*-coumaric acid, protocatechuic acid, syringic acid, sarsasapogenin, diosgenin, smilagenin and trichloroacetic acid were purchased from Sigma-Aldrich Quimica (Madrid, Spain). Isorhamnetin-3-*O*-rutinoside (narcissin) was purchased from Extrasynthese (Genay, France). Protodioscin (97% purity) and shatavarin (98.6% purity) were purchased from Chromadex Chemical Co. (Barcelona, Spain). All cell culture reagents were purchased from Gibco (Madrid, Spain). The extraction solvent ethyl acetate was obtained from Romil Ltd. (Waterbeach, UK). Pure deionized water was obtained from a Milli-Q50 system (Millipore Corporation, Bedford, MA, USA).

### 3.2. Plant Material

*A. acutifolius* L. plants were collected in January 2015 from Borj Cedria (22 km north Tunis). The plant materials were identified and authenticated by Mr. Mounir Kassri and voucher specimens (PLM 53) were deposited at the Herbarium of the Department of Biology, Faculty of Science of Tunis, Tunisia.

Samples of the leaf, stem, pericarp (fruits without seeds) and rhizome (minimum 1 Kg fresh weight each) of *A. acutifolius* L. were cleaned and washed thoroughly with distilled water. Each organ was then left at room temperature in the dark until dryness, and ground to a fine powder in a Mettler AE 200 (Dangoumau type) grinder. All samples were stored at −20 °C until analysis.

### 3.3. Preparation of Ethanolic Extract

Ethanolic extraction was performed using ethanol:water (80:20, *v*/*v*) in a ratio 1:40 (*w*/*v*). The mixture was homogenized in an Ultraturrax (Ultra-Turrax T25, Janke & Kunkel/IKA Labortechnik, Munich, Germany) for 1 min at maximum speed and filtered through filter paper. The residue was extracted again in the same conditions. Ethanolic extracts were pooled together and stored at −20 °C until analysis. All extractions were made in duplicate.

### 3.4. Analysis of Phenolic Compounds

#### 3.4.1. Extraction and Quantification of Free Phenolics

The extraction methods were adapted from Weidner et al. [40] and Sosulski et al. [41] with the following modifications: 20 mL of 80% ethanolic extracts containing soluble phenolics were concentrated to dryness using a rotatory evaporator at 35 °C and 5 mL of distilled water were added. These aqueous solutions were adjusted to pH 2 and filtered through Whatman paper no. 1. The sample was extracted three times with ethyl acetate. The ethyl acetate phase was dried using a rotavapor and dissolved in 1 mL of 96% ethanol. The final extracts were stored at −18 °C prior to analysis.

A Jasco-LC-Net II ADC (Jasco, Madrid, Spain) liquid chromatograph system equipped with a DAD was used. Separation was carried out in a Mediterranea Sea C18 reverse-phase analytical column (25 cm length × 4.6 mm i.d., 5 µm particle size; Teknokroma, Barcelona, Spain). An elution gradient was used with solvents A (water with 1% formic acid) and B (acetonitrile with 1% formic acid): the proportion of solvent B was increased from 0% to 15% in 10 min, then maintained at 15% for 5 min, then raised to 20% over the next 10 min, maintained at 20% for 5 min, and raised to 100% over the next 5 min, maintained at 100% B for 5 min and finally returned to the initial conditions over the following 5 min. The column end was connected directly to a diode array detector (DAD) (Waters 996, Millipore, Manchester, UK). Spectra from all peaks were recorded in the 200–600 nm range and the chromatograms were acquired and quantified at 280 nm.

Stock standard solutions of each compound were prepared by dissolving 10 mg of each analytical standard in 10 mL of 80% ethanol. All solutions were stored at 4 °C. An intermediate solution containing all standard compounds (62.5 μg/mL) was prepared in 80% ethanol, and dilutions from this solution were made at different levels for calibration curves between 0 and 250.

#### 3.4.2. Analysis and Quantification of Flavonoids by HPLC–DAD

Flavonoids were quantified directly from the ethanolic extracts. A Jasco-LC-Net II ADC liquid chromatograph system equipped with a DAD was used. Separation was carried out in a Mediterranea Sea C18 reverse-phase analytical column (25 cm length × 4.6 mm i.d., 5 µm particle size; Teknokroma, Barcelona, Spain). A gradient of solvent A (water with 1% formic acid) and solvent B (acetonitrile with 1% formic acid) was used: the proportion of solvent B was increased from 0% to 20% in 20 min, then to 21% over the next 8 min, maintained at 21% for 2 min, then to 30% over the next 10 min, and to 100% over the next 5 min, maintained at 100% B for 5 min and finally returned to the initial conditions over the next 5 min. The flow rate was 1 mL/min and the column temperature was 30 °C. Spectra from all peaks were recorded in the 200–600 nm range and the chromatograms were acquired at 360 nm for flavonoid glycosides and 370 nm for their aglycones.

The quantification of flavonoids was carried out as described by Fuentes-Alventosa et al. [42]. The identification of individual flavonoid glycosides was based on their retention times (tR) and spectroscopic data. The quantification of individual flavonoid monoglycosides and flavonoid diglycosides was directly performed by HPLC-DAD using an eight-point regression curve in the range of 0–250 μg on the basis of standards. Results were calculated from the mean of three replicates.

### 3.5. Analysis of Saponins

#### 3.5.1. Characterization and Quantification of Saponins by LC-MS

The evaluation of the saponin content was carried out as described by Vázquez-Castilla et al. [25]. An HPLC Waters Alliance (Manchester, UK) system fitted to a Mediterranean Sea C18 reverse-phase analytical column (25 cm length × 4.6 mm i.d., 5 μm particle size; Teknokroma, Barcelona) was used. An elution gradient was used with solvents A (water with 1% formic acid) and B (acetonitrile with 1% formic acid): 0–30 min, 20% B; 30–60 min, linear gradient to 30% B; 60 to 70 min, linear gradient to 100% B; and 70–80 min, linear gradient to 20% B. Saponins were detected using an online connected quadrupole mass analyzer (ZMD4, Micromass, Waters, Inc., Manchester, UK) the flow in the MS was regulated using a split (flow 0.3 mL/min). ESI mass spectra were obtained at ionization energies of 50 and 100 eV (negative mode) and 50 eV (positive mode) with scans from *m*/*z* 200 to 1200. The capillary voltage was 3 kV; the desolvation temperature was 200 °C; the source temperature was 100 °C and the extractor voltage was 12 V.

Two different external standards were used: protodioscin and shatavarin IV. For each standard, 10 dilutions from 0 to 500 μg/mL were prepared and injected into the LC−MS system. For each standard, the selected ion chromatogram corresponding to its molecular ion in negative mode at 100 eV was integrated and the peak area was plotted against the concentration and subjected to regression analysis.

#### 3.5.2. Determination of Exact Mass

For the exact mass determination saponin extract was injected in a high resolution LC/MS system. The liquid chromatograph was Dionex Ultimate 3000 HPLC (Thermo Fisher Scientific, Waltham, MA, USA). Chromatographic separation was achieved in the same conditions than low resolution LC-MS (previous section). A split post-column of 0.4 mL/min was introduced directly on the mass spectrometer ion source. Mass spectrometry was performed using a micrOTOF-QII High Resolution Time-of-Flight mass spectrometer (UHR-TOF) with qQ-TOF geometry (Bruker Daltonics, Bremen, Germany) equipped with an ESI. The instrument was operated in negative ion mode using a scan range of *m*/*z* 50–1200. Mass spectra were acquired through broad-band Collision Induced Dissociation bbCID mode, providing MS and MS/MS spectra, simultaneously. The instrument control was performed using Bruker Daltonics HyStar 3.2.

#### 3.5.3. Preparation of Saponin Extract and Purification of Individual Saponins

The dried ethanolic extract of the different plant parts of *A. acutifolius* was dissolved in water and loaded onto a column of Amberlite XAD-16. The column was washed with water followed by 20%, 40% and 96% ethanol, using a ratio of 1/4 (*v*/*v*) versus the loaded sample. The saponins were eluted with 40% ethanol. The saponin fraction was repetitively subjected to preparative scale reverse phase HPLC, on an ODS column (Shimadzu Prep K, 2.0 × 25.0 m), eluted with acetonitrile:water (1:1 *v*/*v*) at a flow rate of 20 mL/min.

#### 3.5.4. Hydrolysis of Saponins

One hundred µg of each purified saponin and 1 mg of total saponin fractions were separately treated with 2 mL of 1 M H_2_SO_4_ in 70% 2-propanol for 2 h at 100 °C. Cholesterol (50 µg) was added as internal standard. Three mL of water were then added. Aglycones were extracted 3 times with 2 mL dichloromethane (CH_2_Cl_2_). The organic solution was treated 2 times with 1 mL NH_4_OH 1 M and the solvent was eliminated. The CH_2_Cl_2_ extracts were evaporated to dryness in a rotary evaporator. The obtained aglycones were dissolved in CH_2_Cl_2_ (200 µL) and analyzed by gas chromatograph (GC) (Agilent technologies 7820A) coupled to a mass spectrometer (MS) (Agilent technologies 5977E). The column used was an Agilent HP-5MS capillary column (30 m × 0.25 mm i.d., 0.25 μm phase thickness). Helium was used as carrier gas at 1 mL/min. The injector temperature was 260 °C, and the mass spectrometer ion source and interface temperatures were 230 and 280 °C, respectively. The injection volumes (1 μL) were performed in splitless mode. The samples were injected direct (30 s) at an initial oven temperature of 200 °C. After 1 min, the temperature rose at 10 °C/min to 270 °C for 8 min and then at 1 °C/min to 290 °C. The column was held at 290 °C for 28 min. The mass spectra were obtained by electronic impact at 70 eV. Identification of compounds was performed according to those of pure commercial compounds.

The glycosidic part of the saponin was hydrolyzed with trifluoroacetic acid [43] and the released sugars analyzed as alditol acetates [44].

### 3.6. Determination of Antioxidant Capacity

The antioxidant capacity was determined according to the method of 2,2-diphenyl-1-picrylhydrazyl (DPPH) [45]: ten µL of extract were added to 190 µL of DPPH^•^ (3.8 mg/50 mL methanol); after 30 min in the dark, the absorbance was measured (in triplicate) at 480 nm. The efficient concentration EC50, which represents the amount of antioxidant necessary to decrease the initial absorbance by 50%, was calculated from a calibration curve by linear regression for each sample. The activity was expressed as millimoles of Trolox equivalent antioxidant capacity (TEAC) per kilogram of dry sample by means of a dose response curve for Trolox.

The method of 2,2-azino-bis (3 ethylbenzothiazoline-6-sulphonic acid) (ABTS) was also applied and the ABTS assay was performed according to the method of Gonçalves et al. [46], with some modifications [47]. Aliquots of 13 μL of each extract and their dilutions were added to 187 μL of the ABTS^•+^ solution in a 96-well microplate in triplicate. For each sample, a blank with ethanol instead of ABTS^•+^ solution was included. A delay of 30 min was programmed into the reader before readings at 414, 655, and 750 nm. The results were expressed in terms of mmol Trolox/Kg of dry sample.

For the determination of ferric reducing antioxidant power, a modification of the Psarra et al. [48] method was applied. FeCl_3_ was employed as oxidant. Fe^2+^ ion produced from the redox reaction formed a colored product with 2,2′-dipyridyl. For the determination of the reducing power of extracts, a microplate reader was used. Ten microliters of each extract and their dilutions, and 10 μL of 6 mM FeCl_3_ in 5 mM citric acid were placed in each microplate well in quadruplicate. For each sample, a blank without FeCl_3_ was included. After dosification, the microplate was incubated for 20 min at 50 °C in an oven. Then, 180 μL of 5 g/L dipyridyl solution in 1.2% trichloroacetic acid were added to each well. Afterward, a delay of 30 min was programmed into the reader before reading at 490 nm. The results were expressed as millimoles of Trolox equivalent (TE) antioxidant capacity per kilogram of dry sample by means of a dose response curve for Trolox.

### 3.7. Cytotoxicity against Cancer Cell Lines

Two cancer cell lines were included in the present study. Human colon cancer HCT-116 and human liver cancer HepG2 cell lines were purchased from the American Type Culture Collection (ATCC, Manassas, VA, USA). Cell culture media were as follows: HCT-116: McCoy’s 5A medium supplemented with 10% heat-inactivated fetal bovine serum (FBS), 100 U/mL penicillin, and 100 µg/mL streptomycin; HepG2: Eagle’s Minimum Essential Medium supplemented with 10% heat-inactivated FBS, 100 U/mL penicillin, and 100 µg/mL streptomycin. All cell lines were cultured at 37 °C and 5% CO_2_ incubators.

Cell viability was assayed based on the ability of live cells to reduce MTT. HCT-116 human colon cancer cells were cultured in 96-well plates in triplicate at a density of 104 cells/well in 200 μL of medium. The cells were grown to 70–80% confluence and then treated with increasing concentrations of plant extracts (ranging from 0 to 600 μg/mL) dissolved in water.

The cells were incubated for 48 h, and then 20 μL of the MTT solution (5 mg/mL in phosphate buffered saline) were added to each well. The cells were incubated for 3 h at 37 °C in a humidified chamber with 5% CO_2_. The supernatant was then sucked off, after which 100 μL of DMSO were added to each well. The culture plate was gently agitated (about 30 min) to make the purple formazan dissolve completely. The concentration of formazan was measured at 490 nm using a Multiskan Spectrum microplate reader (ThermoLab systems, Boston, MA, USA). The IC_50_ values were calculated from the regression curves of % cell viability vs extract concentration for each assay. All MTT assays were carried out in three separate trials, each experiment including five replicate sets.

### 3.8. Pancreatic Lipase Inhibitory Activity Assay In Vitro

Lipase activity was measured using *p*-NPB (*p*-nitrophenyl butyrate) as substrate. The method was modified from the one previously described by Kim et al. [49]. Briefly, an enzyme buffer was prepared by the addition 20 μL of solution of porcine pancreatic lipase (20 mg/mL in TRIS buffer, pH 7) to 160 μL of Tris buffer (100 mM Tris-HCl and 5 mM CaCl2, pH 7.0). Then, increasing concentrations of various extracts (ranging from 0 to 11.25 mg/mL) dissolved in TRIS buffer were mixed with 20 μL of the enzyme buffer and incubated for 30 min at 37 °C. Twenty µL of substrate (10 mM *p*-NPB in dimethyl formamide) were then added. Lipase activity was determined by measuring the hydrolysis of *p*-NPB to p-nitrophenol at 405 nm using an ELISA reader. The inhibition of lipase activity was expressed as the percentage of absorbance decrease when porcine pancreatic lipase was incubated with the test compounds. Lipase inhibition (%) was calculated according the following formula:

Inhibitory activity (I %) = (A − B)/A × 100

where A is the lipase activity in the reaction solution without sample and B is the lipase activity in the reaction solution containing sample. The measurements were made in triplicate and the IC_50_ (inhibitory concentration at which 50% inhibition of enzyme activity occurs) values of the test samples were determined by performing the assay as above with varying concentrations of test samples. The IC_50_ values were determined from the regression curves of inhibition percentage vs extract concentration.

### 3.9. Antimicrobial Activity

#### 3.9.1. Microbial Strains

The antibacterial activities of the ethanolic extracts of *A. acutifolius* L. leaves and rhizomes were tested against 15 clinical bacterial (Gram-positive and Gram-negative) and fungal species isolated from various patients and identified at the Charles Nicolle University Hospital of Tunisia. The Gram-positive bacteria were *Staphylococcus aureus*, *Staphylococcus epidermidis*, *Enterococcus faecalis*, *Enterococcus faecium*, *Listeria monocytogenes* and methicillin resistant *S. aureus* (MR). The Gram-negative bacteria included *Klebsiella pneumoniae*, multidrug resistant *Klebsiella pneumoniae* (MDR), *Salmonella typhimurium*, *Escherichia coli*, *Proteus mirabilis*, *Citrobacter koseri*, multidrug resistant *Pseudomonas aeruginosa* (MDR), *Enterobacter cloacae* and ß-lactamase-producing *E. coli* (ESBL) and the fungal species were *Geotricum capitatum*, *Candida albicans*, *Candida galabrata*, *Candida parapsilosis* and *Candida tropicalis*. All isolates were identified by cultural, morphological, and biochemical tests. All the strains tested were kept at 4 °C in Mueller–Hinton Agar for bacteria and Sabouraud dextrose agar for fungus they were sub-cultured every month.

#### 3.9.2. Well Diffusion Method

The antibacterial activity of organ extracts was assessed by the agar disk diffusion assay [31] against 15 human pathogenic bacteria. Inoculums were prepared with fresh cultures of microbial strains, cultured on Mueller–Hinton broth (MHB) for 5 h at 37 °C. The agar well diffusion method was used for the determination of inhibition zones. One milliliter of inoculums was mixed with 19 mL of Mueller–Hinton agar (the final concentration of bacteria was 10^6^ CFU/mL). After 10 min, wells (d = 6 mm) were made in the Petri plates. Sixty micro-liters of the filter-sterilized ethanolic extracts of the selected plants parts were dropped into each well (at 100 mg/mL). In order to accelerate the diffusion of the ethanolic extracts of the leaves, pericarps and rhizomes into the agar, the plates were incubated at 4 °C for 1 h followed by incubation at 37 °C for 24 h [31]. The results of the diffusion method displayed as zones of growth inhibition were calculated as the mean of 3 experiments. Gentamicin and spectromicin were used as antibacterial and Amphotericin B as antifungal.

For the antifungal activity, five fungal strains were cultured on Sabouraud Dextrose broth (MHB) for 5 h at 37 °C. The agar well diffusion method was used for the determination of inhibition zones. One milliliter of inoculums was mixed with 19 mL of Sabouraud Dextrose agar for (the final concentration of fungal was 106 CFU/mL). After 10 min, wells (d = 6 mm) were made in the Petri plates. Sixty micro-liters of the filter-sterilized ethanolic extracts of the selected plants parts were dropped into each well (at 100 mg/mL). In order to accelerate the diffusion of the ethanolic extracts of the leaves and rhizomes into the agar, the plates were incubated at 4 °C for 1 h followed by incubation at 37 °C for 24 h. As for the antibacterial activity, the antifungal one was evaluated by measuring the diameter of the growth inhibition zone around the well [31]. The susceptibility of the standard was determined using a disc paper containing 20 µg of amphoterecin B. Each experiment was carried out in triplicate and the mean diameter of the inhibition zone was recorded.

#### 3.9.3. MIC and MBC Determinations for Bacteria

Minimum inhibitory concentration (MIC) and minimum bactericidal concentration (MBC) were determined in the Mueller–Hinton broth (MHB), using the 2-fold macro-dilution method in the Mueller–Hinton broth (MHB) as described by the Clinical and Laboratory Standards Institute (CLSI). The final concentration of bacteria in each macro-broth dilution tube was 5 × 10^5^ CFU/mL of MHB. Serial dilutions of filter-sterilized ethanolic extract (100 to 0.195 mg/mL) were used. The MIC was defined as the lowest concentration of sample that resulted in no visible growth after 24 h of incubation at 37 °C. A total of 100 μL from clear tubes was plated onto Mueller–Hinton agar (MHA) plates [31]. The MBC was defined as the lowest concentration of sample that resulted in ≥ 99.9% kill of the initial inoculums.

#### 3.9.4. MIC and MFC Determinations for Fungal

Minimum inhibitory concentration (MIC) and Minimum Fungicidal Concentration (MFC) were determined in the Sabouraud Dextrose Broth (SDB), using the 2-fold macro-dilution method in the the Sabouraud Dextrose Broth (SDB) as described by the Clinical and Laboratory Standards Institute (CLSI). The final concentration of fungal in each macro-broth dilution tube was 5 × 105 CFU/mL of SDB. Serial dilutions of filter-sterilized ethanolic extract (100 to 0.195 mg/mL) were used. The MIC was defined as the lowest concentration of sample that resulted in no visible growth after 24 h of incubation at 37 °C. A total of 100 μL from clear tubes was plated onto the Sabouraud Dextrose Broth (SDB) plates [31]. The minimum fungicidal concentration was regarded as the lowest extract concentration that hindered visible growth of the subculture (did not yield any fungal growth on the solid medium used).

### 3.10. Statistical Analysis

All data presented are the mean ± standard errors from three measurements. Comparisons of means were determined by one-way ANOVA, followed by the Duncan Test. Differences were considered significant at *p* < 0.05. The Statistic 7.0 package was used.

## 4. Conclusions

The present study showed, for the first time, the bioactive composition of different *A. acutifolius* organs and their antioxidant, cytotoxic, lipase inhibitory and antimicrobial properties. The results reveal that the stem from this plant contains high amounts of total flavonoids and simple phenolics. Caffeic acid was the major phenolic acid in the ethanolic extracts of the different plant parts of *A. acutifolius*, followed by significant amounts of ferulic, p-hydroxybenzoic and protocatechuic acids. Flavonoids were detected in all the organs, but their glycosides were only found in the stems. The distribution of saponins varied greatly among *A. acutifolius* plant parts, with the highest amount being found in the rhizome extract followed by the pericarp, leaf and stem. Stem extracts showed the best antioxidant activity in ferric reducing antioxidant power, and DPPH radical assays. However, the pericarp had the best antioxidant activity in ABTS radical assays. Moreover, the rhizome and leaf extracts exhibited significant cytotoxicity against two carcinoma cell lines (colon cancer HCT-116 and liver cancer HepG2). The pericarp, leaf and rhizome showed good lipase inhibitory activity. The leaf and rhizome extracts were screened for their antimicrobial activity against human bacterial and yeast isolates and the result showed that the leaf extracts exhibited a high inhibition against all the bacteria and yeast tested.

Our study provides valuable evidence that several unused parts of *A. acutifolius* plants could be great sources of bioactive phytochemicals. These findings also support the proposal that this plant could have great potential as ingredients for functional foods, as they possess protective functions against oxidant-induced damage, obesity, different kinds of cancer, bacteria and yeast. As saponins were the major compounds in the different plant parts of *A. acutifolius*, further work is needed including the structural characterization of purified saponins obtained in this study, as well as their specific cytotoxic activity against different human cancer cell types.

## Figures and Tables

**Table 1:** Flavonoids and simple phenolic compositions of different plant part extracts from *A. acutifolius.*

Phenolics	Leaf	Stem	Pericarp	Rhizome
Gallic acid	36.63 ± 0.54 a	nd	36.24 ± 0.07 a	36.81 ± 0.73 a
Protocatechuic acid	14.62 ± 0.06 a	25.92 ± 0.27 d	18.63 ± 0.07 c	16.51 ± 0.06 b
*p*-hydroxybenzoic acid	23.51 ± 0.04 b	15.26 ± 0.09 a	25.05 ± 0.10 c	36.72 ± 0.18 d
Vanillic acid	8.94 ± 0.07 b	10.61 ± 0.05 c	3.70 ± 0.10 a	41.11 ± 0.02 d
Caffeic acid	39.42 ± 0.11 a	297.05 ± 5.89 d	67.78 ± 0.33 c	48.82 ± 0.26 b
*p*-hydroxybenzaldehyde	11.36 ± 0.09 b	9.97 ± 0.22 a	12.26 ± 0.14 c	17.67 ± 0.09 d
Syringic acid	9.36 ± 0.05 b	0.67 ± 0.14 a	nd	37.98 ± 0.20 c
Vanillin	13.95 ± 0.09 b	12.07 ± 0.05 a	nd	12.76 ± 0.65 a
*p*-Coumaric acid	2.95 ± 0.24 a	6.79 ± 0.13 b	13.58 ± 0.10 c	15.34 ± 0.12 d
t-ferulic acid	34.73 ± 0.11 c	27.53 ± 0.02 a	30.06 ± 0.11 b	34.90 ± 0.17 c
t-Sinapic acid	9.75 ± 0.11 b	9.11 ± 0.04 a	9.59 ± 0.22 b	13.17 ± 0.05 c
Rutin	nd	316.27 ± 31.63	nd	nd
Narcissin	nd	154.10 ± 6. 86	nd	nd
Quercetin	nd	nd	9.08 ± 0.20 a	9.60 ± 0.09 b
Naringenin	22.89 ± 0.29 c	61.27 ± 0.09 d	1.51 ± 0.05 a	4.88 ± 0.34 b
Kaempferol	22.08 ± 0.22 b	4.94 ± 0.15 a	nd	27.06 ± 0.11 c
Total simple phenolics	205.23 ± 1.28 a	414.99 ± 6.90 d	216.88 ± 1.25 b	311.80 ± 1.23 c
Total flavonoids	44.98 ± 0.51 c	536.55 ± 25.01 d	10.59 ± 0.26 a	41.54 ± 0.54 b
Total phenolics	250.20 ± 1.79 b	951.54 ± 31.91 d	227.47 ± 1.50 a	353.34 ± 1.77 c

Data mg/kg dry leaves, pericarp, stem and rhizome are the mean of three replicates. Results correspond to the mean ± standard deviation. Different letters within the same row (a–d) mean that there are significant differences (*p* < 0.05). nd, not detected.

**Table 2:** Characterization of the saponins tentatively identified by LC-MS (+/−) of the ethanolic extracts of different organs from A. *acutifolius*.

Molecular Ion (*m*/*z*)						Ion Fragmentation	
Saponin	Rt ^a^ (min)	Formula	[M − H]-Experimental	[M − H]- Theoretical	Error (ppm)	Neg Mode	Pos Mode
HTSAP-1	24.9	C_50_H_83_O_23_	1051.5331	1051.5284	4.4	^b^ Pen919-^c^ Hex757-Hex595-Hex433	[-Na-H2O]1035-Hex873-Hex711-Pen579-Hex417 ^e^
ACSAP-1	27.8	C_50_H_83_O_23_	1051.5331	1051.5306	2.4	Pen919-Hex757-Hex595-Hex433	[-Na-H2O]1035-Hex873-Hex711-Pen579-Hex417
HTSAP-3	29.6	C_49_H_83_O_22_	1021.5225	1021.5212	1.2	Pen889-Pen757-Hex595-Hex433	[-Na-H2O]1005-Pen873-Hex711-Pen579-Hex417
HTSAP-6	32.9	C_50_H_83_O_23_	1035.5381	1035.5383	0.1	Pen903-^d^ DoHex757-Hex595-Hex433 ^e^	[-Na-H2O]1019-DoHex873-Pen741-Hex579-Hex417
ACSAP-2	42.2	C_44_H_71_O_18_	887.4646	887.4601	5	Pen755-Hex593-Hex431 ^e^	[-Na-H2O]871-Hex709-Pen577-Hex415 ^e^
ACSAP-3	44.1	C_43_H_69_O_17_	857.4540	857.4503	4.3	Pen725-Pen593-Hex431	[-Na-H2O]841-Pen709-Pen577-Hex415

^a^ Rt: retention time. ^b^ Pen: Pentose. ^c^ Hex: Hexose. ^d^ DoHex: Deoxyhexose. ^e^ 433,431,417,415: Genins.

**Table 3:** Saponins identified in the leaf, stem, pericarp and rhizome ethanolic extracts of *A. acutifolius*, calculated from the molecular ion of the peak area obtained by HPLC-MS.

Saponin	Leaf	Stem	Pericarp	Rhizome
HTSAP-1	530.4 ± 8.1 7%	254.3 ± 27.6 18%	215.5 ± 4.5 2%	8355.0 ± 607.8 24%
ACSAP-1	882.1 ± 37.6 12%	220.0 ± 2.7 16%	2988.3 ± 132.1 25%	nd
HTSAP-3	353.7 ± 2.3 5%	96.3 ± 5.6 7%	509.9 ± 51.5 4%	16,273.6 ± 493.0 47%
HTSAP-6	5354.1 ± 10.8 75%	899.5 ± 48.2 63%	6935.1 ± 348.3 59%	7169.2 ± 157.7 21%
ACSAP-2	nd	nd	nd	928.9 ± 39.6 3%
ACSAP-3	nd	nd	nd	1704.02 ± 134.3 5%
Total saponins	7120.4 ± 20.9 b	1418.6 ± 72.9 a	10,648.7 ± 563.4 c	34,430.8 ± 1353.2 d

Data mg/kg dry leaves, pericarp, stem and rhizome are the mean of three replicates. Results correspond to the mean ± standard deviation of three replicates. Different letters within the same row (a–d) mean that there are significant differences (*p* < 0.05). nd: not detected.

**Table 4:** Antibacterial and antifungal activities of leaf and rhizome ethanol extracts of *A. acutifolius.*

Zone of Inhibition (mm)				
Bacteria (Clinical Isolate)	Ethanolic Extract		Standard Antibiotics	
	Leaf	Rhizome	Gent	Strepto
Gram+				
*Staphylococcus aureus*	16 ± 4.3 a	0 ± 0	26 ± 1.6 c	21 ± 0.0 b
Methicillin resistant *Staphylococcus aureus* (MR)	18 ± 1.6 c	13 ± 1.2 a	16 ± 1.6 b	21 ± 0.0 d
*Staphylococcus epidermidis*	14 ± 2.8 a	13 ± 1.4 a	36 ± 0.0 c	25 ± 0.0 b
*Enterococcus faecalis*	17 ± 2.8 c	14 ± 4.3 b	12 ± 0.0 a	0 ± 0
*Enterococcus faecium*	17 ± 7.1 a	20 ± 7.1 b	45 ± 0.0 d	30 ± 0.0 c
*Listeria monocytogenes*	17 ± 3.2 c	0 ± 0	14 ± 0.0 b	12 ± 0.0 a
Gram-				
*Klebsiella pneumonia*	14 ± 2.8 b	11 ± 00 a	35 ± 0.0 d	30 ± 0.0 c
Multidrug resistant *Klebsiella pneumoniae* (MDR)	18 ± 2.8 b	12 ± 1.6 a	21± 0.0 c	0 ± 0
*Salmonella typhimurium*	22 ± 4.3 c	12 ±1.6 a	26 ± 0.0 d	16 ± 0.0 b
*Escherichia coli*	17 ± 8.5	11 ±1.6	20 ± 0.0	12 ± 0.0
Extended spectrum ß-lactamase producing (ESBL) *Escherichia coli*	15 ± 4.3 b	0 ± 0	10 ± 1.6 a	0 ± 0
*Enterobacter cloacae*	18 ± 5.8 c	11 ± 0 a	22 ± 0.0 d	16 ± 0.0 b
Multidrug resistant *Pseudomonas aeruginosa* (MDR)	16 ± 3.2 b	14 ± 4.3 a	22 ± 0.0 c	14 ± 0.0 a
*Citrobacter koseri*	16 ± 5.6 b	12 ± 1.6 a	24 ± 0.0 d	18 ± 0.0 c
*Proteus mirabilis*	14 ± 3.2 a	0 ± 0	30 ± 0.0 b	0 ± 0
Fungal		Amph B		
*Geotricum capitatum*	26 ± 3.2 c	16 ± 7.1 a	22 ± 0.0 b	
*Cándida albicans*	13 ± 1.6 b	13 ± 1.6 b	11 ± 0.0 a	
*Cándida galabrata*	15 ± 2.8 a	19 ± 3.2 b	14 ± 0.0 a	
*Cándida parapsilosis*	27 ± 4.3 b	26 ± 7.1 b	18 ± 0.0 a	
*Cándida tropicalis*	21 ± 9 b	21 ± 1.6 b	12 ± 0.0 a	

Results correspond to the mean ± standard deviation of three replicates. Different letters within the same row mean that there are significant differences (*p* < 0.05). Gent: gentamicine. Strepto: streptomicin, Amph B: amphotericine B. Diameter of disc used is equal to 6 mm.

**Table 5:** Minimal inhibition concentrations (MICs), minimal bactericidal concentrations (MBCs) (mg/mL) and minimal fungicidal concentration (MFC) of selected extracts of *A. acutifolius* against bacteria and fungi.

Bacteria (Clinical Isolate)	Ethanolic Extract			
	Leaf		Rhizome	
Gram+	MIC	MBC	MIC	MBC
*Staphylococcus aureus*	0.78	12.5	-	-
Methicillin resistant *Staphylococcus aureus* (MR)	0.39	6.25	3.12	50
*Staphylococcus epidermidis*	1.56	25	3.12	50
*Enterococcus faecalis*	0.39	6.25	1.56	25
*Enterococcus faecium*	0.39	6.25	0.195	3.125
*Listeria monocytogenes*	0.39	6.25	-	-
Gram-
*Klebsiella pneumonia*	1.56	25	3.12	50
Multidrug resistant (MDR) *Klebsiella pneumonia*	0.39	6.25	3.12	50
*Salmonella typhimurium*	0.195	3.125	3.12	50
*Escherichia coli*	0.39	6.25	3.12	50
Extended spectrum ß-lactamase producing (ESBL) *Escherichia coli*	1.56	25	-	-
*Enterobacter cloacae*	0.39	6.25	3.12	50
Multidrug resistant *Pseu-domonas aeruginosa (MDR)*	0.78	12.5	1.56	25
*Citrobacter koseri*	0.78	12.5	3.12	50
*Proteus mirabilis*	1.56	25	-	-
Fungal	MIC	MFC	MIC	MFC
*Geotricum capitatum*	0.096	0.195	0.195	0.390
*Cándida albicans*	0.195	0.390	0.195	0.390
*Cándida galabrata*	0.195	0.390	0.195	0.390
*Cándida parapsilosis*	0.096	0.195	0.096	0.195
*Cándida tropicalis*	0.195	0.390	0.195	0.390

## Sample Availability

Samples of the compounds are available from the authors.
